# Characteristics of EEG activity in soccer players’ alerting effects under different cues

**DOI:** 10.3389/fpsyg.2026.1918092

**Published:** 2026-08-21

**Authors:** Chuikun Li, Cuilan Wei, Shuai Wu, Yeting Zhang, Qiongjia Yuan

**Affiliations:** 1 College of Physical Education, Chengdu University, Chengdu, China; 2 College of Physical Education, Chengdu University of Technology, Chengdu, China; 3 College of Physical Education, China West Normal University, Nanchong, China; 4 College of Aviation Physical Education, Civil Aviation Flight University of China, Guanghan, China; 5 School of Sports Medicine and Health, Chengdu Sport University, Chengdu, China

**Keywords:** alerting, EEG, N1, P2, soccer players

## Abstract

Previous studies have found a positive correlation between athletic expertise and the alerting effect; however, few studies have investigated these associations among soccer players. This study employed an alerting task to compare the alerting effect between elite soccer players and novices. We report an ERP study involving 68 participants, which allows us to reexamine the mechanisms underlying alerting. We observed that elite athletes had significantly shorter reaction times on both dual-cue and no-cue trials compared to novices, and found that elite athletes’ alerting network test scores were significantly higher. Elite soccer players generally exhibited faster reactions, and athletic expertise was positively correlated with cognitive performance. Further analysis revealed that, for elite athletes, the N1 peak amplitude evoked by dual-cue conditions in the central, parietal, parieto-occipital, and occipital regions was larger than that of novices; a similar pattern was observed in the central region for no-cue conditions. For dual-cue conditions, the parietal, parieto-occipital, and occipital regions exhibited smaller N1 peak amplitudes; no between-group differences were found in N1 latency. It was also found that, for the dual-cue condition, elite athletes exhibited smaller P2 peak amplitudes in the frontal, fronto-central, and central regions compared to novices. For the dual-cue condition, the prefrontal, frontal, fronto-central, and central regions exhibited larger P2 peak amplitudes. Additionally, the P2 latency for the dual-cue condition was earlier than for the no-cue condition, though no intergroup differences were observed. The results indicate that elite soccer players exhibit significant behavioral and neurophysiological advantages, further demonstrating that the enhancement of N1 and the attenuation of P2 are associated with soccer skill level. Elite soccer players exhibited larger N1 amplitudes and smaller P2 amplitudes in response to both dual-cue and no-cue stimuli. In summary, this study confirms that elite soccer players possess dual advantages in alerting function, providing new empirical evidence for understanding the neuroplasticity mechanisms by which long-term athletic training shapes cognitive function.

## 1 Introduction

The alerting network is a core component of the attentional system, enabling individuals to maintain a state of sensitivity to potential stimuli in their environment and respond rapidly (Posner and Petersen, 1990). The alerting functions of elite athletes (e.g., reaction time, signal detection sensitivity) are significantly positively correlated with athletic performance (e.g., predictive accuracy, decision-making speed) (Ballester et al., 2018; Hüttermann and Memmert, 2018). Soccer players must maintain a high level of vigilance throughout lengthy matches to detect sudden threats or opportunities in real time and execute immediate motor responses. Among the three subsystems of the attentional network, although orientation and executive control are also involved in soccer performance (Verburgh et al., 2014), the vigilance network plays a more fundamental role in soccer cognition. Soccer matches involve frequent changes of direction (2–4 s per change), continuous environmental scanning, and intermittent sprints, constituting a sustained rather than sporadic cognitive load (Aarons et al., 2023), and the state of reaction readiness maintained by the vigilance network is precisely the fundamental requirement throughout the entire game (Klösch et al., 2022).

Soccer serves as an ideal model for examining the alerting network due to the high alignment between its perceptual-cognitive characteristics and alerting functions. In a dynamic environment, with 22 players and constant shifts in ball possession, athletes must continuously monitor highly uncertain scenarios (Mann et al., 2007); they must perform wide-range visual scans at a frequency of 3–5 times per second both before and after gaining possession, while simultaneously tracking the relative positions of multiple players (Jordet, 2005); passing directions and defensive movements are highly variable, making it difficult to effectively predict the spatiotemporal timing of key events (Araújo et al., 2006); The available window from a change in possession to effective decision-making is often less than 500 milliseconds, requiring perceptual-motor coupling under extreme time pressure (Roca et al., 2012). These characteristics subject soccer players to a high-intensity, sustained vigilance load that directly corresponds to their core functions, making them an ideal group for examining the plasticity of alerting networks (Voss et al., 2010). In contrast, sports such as track and field feature structured, highly predictable environments and lower vigilance loads; while sports like basketball share some similarities, the intervals between plays provide a buffer that mitigates the intensity of sustained vigilance (Nuri et al., 2013). The combined intensity of soccer—in terms of dynamism, unpredictability, and time pressure—provides a unique cognitive window for vigilance research. Recent cognitive computing studies have revealed that soccer players’ advantages in psychomotor alerting tasks stem primarily from shorter non-decision times (information encoding and action generation), rather than improved decision quality (Zhong et al., 2025), indicating that vigilance confers a cognitive dividend in the temporal dimension. Neuroimaging further reveals that their level of alertness is specifically associated with the structural and functional connectivity of multiple subregions of the thalamus (the right ventrolateral thalamus and the left intraparietal nucleus) (Zhong et al., 2025), providing biological evidence supporting the neuroplastic association between soccer experience and the alerting network.

Recent studies have shown that electroencephalography (EEG) and its derivative, event-related potentials (ERPs), can serve as neuroimaging tools for motor actions (Cheron et al., 2016; Ligeza et al., 2018; Park et al., 2015; Wang et al., 2019). Among ERP components, the N1 is a core indicator closely associated with the alerting network (Hillyard et al., 1973; Mangun and Hillyard, 1991). It has a latency of approximately 80–150 milliseconds, with peak amplitude in the posterior and temporo-parietal regions, and exhibits a larger (more negative) amplitude in tasks involving rapid attentional orientation and the detection of vigilance signals (Vogel and Luck, 2000; Eimer, 1994). In alerting tasks, the N1 evoked by valid cues is significantly larger (more negative) than that evoked by invalid cues; this effect is thought to reflect an early perceptual gain for target stimuli and is typically interpreted as evidence of alerting network activation, specifically the rapid detection of sudden targets (Posner and Petersen, 1990; Fan et al., 2005). Overall, the enhancement of N1 amplitude in alerting tasks is viewed as an expression of the attentional system’s sensitization to anticipated locations or time windows, thereby facilitating rapid responses (Hillyard and Anllo-Vento, 1998; Natale et al., 2006). However, current electrophysiological evidence regarding enhanced alerting network function in elite athletes presents a complex picture due to differences in task paradigms and sports disciplines. Studies using passive or simple detection tasks have generally failed to observe a consistent advantage in N1 amplitude among athletes; for example, Lin et al. (2021), who found in a visual detection task that while soccer players exhibited a significant shortening of the N1 latency (reflecting faster early perceptual processing), their N1 amplitude did not differ significantly from that of the control group, suggesting that athletes’ alerting advantage may be reflected more in processing speed than in gains in the allocation of attentional resources. In contrast, when tasks place higher demands on sustained monitoring and response inhibition, the N1 modulation effect becomes more pronounced. Chen et al. (2023) employed a Go/Nogo dual-task paradigm combined with postural control. They found that skilled athletes (including those in gymnastics, martial arts, and other disciplines) exhibited significantly higher N1 amplitudes than non-athletes, indicating that athletes can flexibly allocate early sensory gains in situations requiring the maintenance of high alertness while simultaneously inhibiting irrelevant responses. These findings suggest that the sensitivity of N1 amplitude to arousal regulation may be modulated by cognitive task demands, particularly the influence of sustained vigilance load and response inhibition pressure (Neuhaus et al., 2010; Galvao-Carmona et al., 2014). Specifically, when a task requires only brief alertness preparation, the early perceptual gain in athletes may not be significant; however, when a task demands sustained target monitoring and rapid discrimination, the mobilization of resources in the alerting network is more readily reflected in N1 amplitude. Therefore, differences in the cognitive demands of task contexts may be a key factor contributing to the inconsistent findings regarding alertness-related N1 effects across studies. Future research should systematically manipulate the level of alerting demands (e.g., cue validity, target occurrence frequency, response complexity) within a single experimental framework, or conduct cross-task comparisons among athletes from different sports, to further clarify the boundary conditions of athletes’ alerting advantages and their neurophysiological mechanisms.

Furthermore, ERP is known for its high sensitivity to millisecond-level temporal dynamics between stimulus presentation and response execution and is considered to reflect implicit and unique cognitive processes characterized by specific components. P2 (or P200) is regarded as one of the key components of the alerting network, primarily associated with early attention allocation and stimulus perceptual processing, reflecting rapid detection of task-relevant stimuli and response preparation (Luck and Hillyard, 1994; Thorpe et al., 1996; Carretié et al., 2001). The P2 typically manifests as a positive wave in the fronto-central or anterior regions, peaking between approximately 150 and 250 milliseconds after stimulus onset, and is closely associated with attentional tuning and sensory gating processes during a state of alertness (Crowley and Colrain, 2004; Freunberger et al., 2007). Specifically, P2 amplitude is sensitive to the physical properties of the stimulus and early attentional resource allocation, while P2 latency is related to the speed of early stimulus categorization (Okita, 1979; Luck, 2014). In alerting tasks (such as sustained attention or cue-target paradigms), an increase in the amplitude of the motion-induced P2 is thought to reflect early perceptual gains and heightened vigilance toward valid cues or salient targets (Sanchez-Lopez et al., 2016; Jin et al., 2011). It should be noted that this study did not include the P3 component in the analytical framework of the alerting network. Although P3 (particularly P3b) is often associated with processes such as the allocation of attentional resources, situational updating, and working memory updating, its functional role is more focused on late-stage cognitive processing following stimulus evaluation and classification, rather than the maintenance of the alerting state itself or rapid detection processes (Polich, 2007; Nieuwenhuis et al., 2005). Existing evidence suggests that the P3 is relatively sensitive to variables such as task difficulty, response decisions, and subjective probability; however, changes in its latency and amplitude are not core indicators of the alerting network, and there is a lack of a stable and independent correspondence between these changes and the alerting effect (Nieuwenhuis et al., 2005; Sarter et al., 2001). Therefore, the P3 is not suitable as a direct electrophysiological marker reflecting the function of the alerting network and is thus not discussed in this study, which focuses on the framework of early perception and rapid detection mechanisms. Therefore, the P3 is not suitable as a direct electrophysiological marker reflecting the function of the alerting network and is thus not discussed in this study, which focuses on the framework of early perception and rapid detection mechanisms. In summary, within the research context of sustained attention and rapid detection, analyzing the alerting network in conjunction with the specific activity of the P2 component helps deepen our understanding of the cognitive characteristics associated with soccer expertise and provides a valuable complement to the evidence offered by the N1 component.

This study aims to thoroughly investigate the distinctive features of attentional function in elite soccer players by utilizing alerting tasks (such as the cue-target paradigm) and combining behavioral reaction times with electrophysiological measures of alerting ERP components (N1, P2), with the goal of deepening our understanding of sport-specific cognitive expertise in soccer. The specific research question is: When faced with stimuli requiring sustained monitoring and rapid detection, do elite soccer players exhibit advantages in early perceptual gain and rapid response efficiency, specifically manifested as shorter reaction times and enhanced modulation of alerting-related ERP components? Based on this, the following research hypotheses are proposed: Hypothesis 1: The alerting network function of the elite group is superior to that of the novice group, as evidenced by significantly shorter reaction times. Hypothesis 2: Under both dual-cue and no-cue conditions, differences will exist between the elite and novice groups in the N1 and P2 components. Specifically, the N1 amplitude in the elite group will be significantly greater than that in the novice group, reflecting greater efficiency in early perceptual gain; simultaneously, the P2 amplitude in the elite group will be significantly smaller than that in the novice group (or the latency will be significantly shorter), reflecting more efficient early classification and response preparation processes for task-relevant stimuli.

## 2 Participants and methods of research

### 2.1 Participants

This study employs a cross-sectional design and uses G*Power 3.1.9.4 software to conduct a prior power analysis (Faul et al., 2007; Dias et al., 2025) to estimate the sample size required for a repeated-measures ANOVA design. Based on an effect size of *f* = 0.20, a significance level of *α* = 0.05, a power level of 0.9, two measurement points, and an assumed correlation of 0.5 between repeated measurements, the total required sample size was 68 participants. Strict adherence to participant inclusion criteria: (i) No history of physical illness, traumatic brain injury, neurological disorders, or mental illnesses; currently in good mental health (General Health Questionnaire-12 scores all < 8) (Shivakumar et al., 2015); no use of neurological and/or psychiatric medications; and no history of alcohol dependence; (ii) All participants must be right-handed; (iii) Participants must have normal visual acuity or corrected visual acuity, with no color blindness or color weakness; (iv) Intelligence (Raven’s Standard Progressive Matrices score ≥ 44) (Raven, 2000) and anxiety levels (Self-Rating Anxiety Scale score < 50) (Zung, 1971). Considering potential data loss due to EEG signal quality issues, a total of 73 volunteers (49 males, 24 females) were recruited and divided into an elite group and a novice group based on their soccer experience. The experimental protocol was approved by the Human Ethics Committee of Chengdu Sport University (Approval No.: (2020) 22) and was conducted in accordance with the ethical standards set forth in the 1975 Declaration of Helsinki (2008 revision). Prior to testing, all participants signed a written informed consent form. Data collection took place from December 23, 2019, to January 15, 2020. Among the participants, 5 subjects had poor-quality EEG data, with artifact removal exceeding 30%; therefore, data from these 5 subjects were excluded from the final analysis. Consequently, the elite athletes group consisted of 34 participants (23 males), all of whom were high-level athletes from a soccer academy. The average training duration for this group was 7.27 years, with a training frequency of at least 4 times per week and a training duration of at least 2 h per session; all participants held National Level 2 or higher athlete certifications; The novice athletes group consisted of 34 participants (23 males), all of whom were college students enrolled in specialized soccer programs with at least 3 months of training experience (see Table 1).

**Table 1:** Demographic information of participants.

Type	Novice	Elite
Number	34	34
Age, mean ± SD, years	20.85 ± 1.31	20.41 ± 1.40
Years of training, mean ± SD	2.49 ± 0.93	7.27 ± 2.03^**^
Training frequency (days/week)	0	No fewer than 4 times
Training time (hours/day)	0	No fewer than 2 h
Level achievement^a^	0	National First Level, *n* = 13 National Second Level, *n* = 21

Compared with novice athletes, ^*^p < 0.05, ^**^p < 0.01.

^a^According to the General Administration of Sport of China, the ranking of athletic categories from lowest to highest is: National Second Level Athlete, National First Level Athlete, and National Elite Athlete.

### 2.2 Research methods

#### 2.2.1 Experimental process

**Figure 1:**
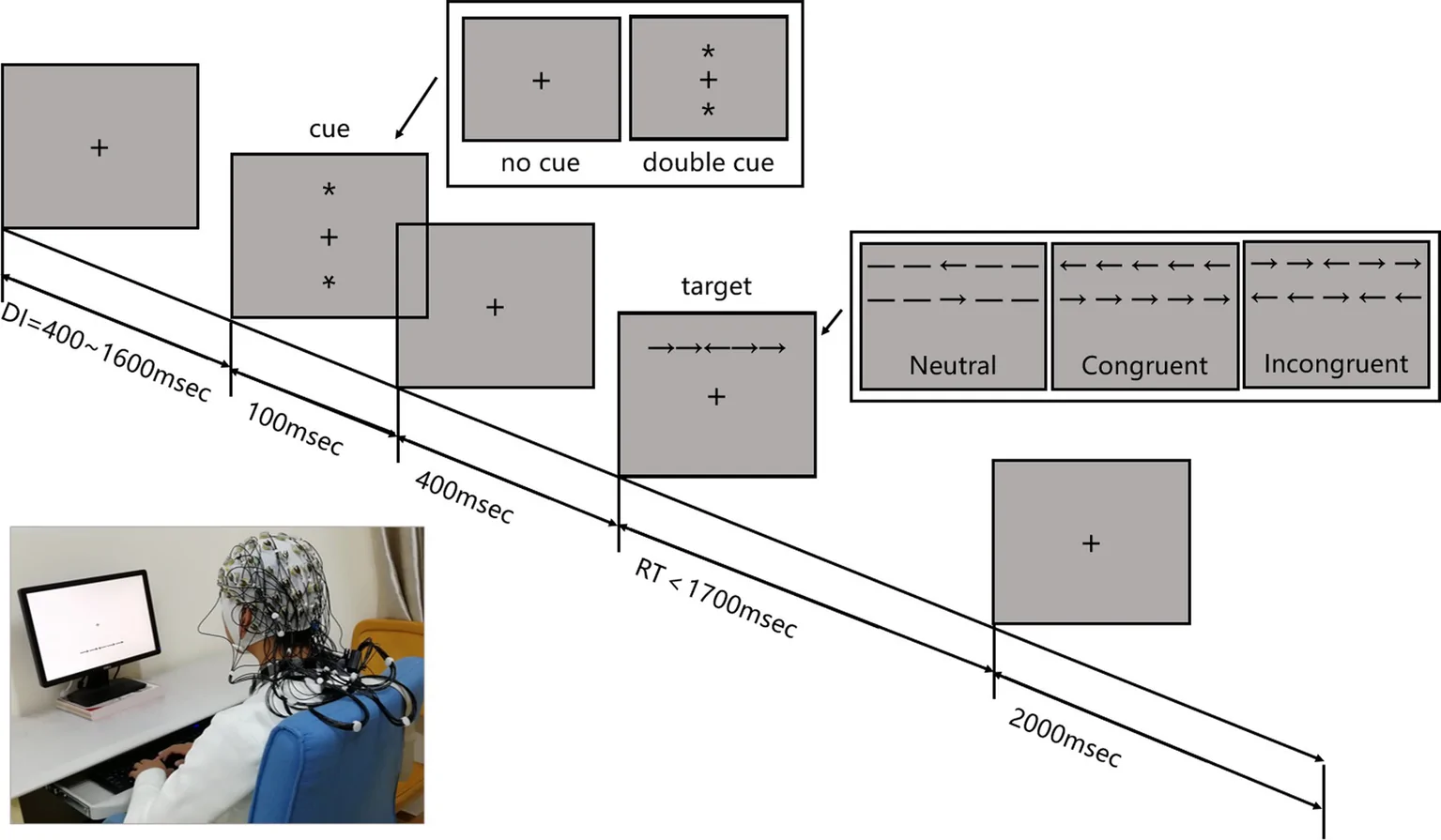
Schematic representation of the alerting network test.

An alerting task was designed in accordance with the research objectives; the specific experimental procedure is shown in Figure 1. Before the experiment, the experimenter briefed the participants on the basic procedure. Participants were instructed to maintain a consistent body posture throughout the test, minimize blinking and swallowing, and avoid electromyographic interference with EEG signals. They were also asked to focus their attention on the stimuli presented on the screen and press the correct keys as quickly as possible. Participants placed both hands on the keyboard, with the index finger of their left hand on the “F” key and the index finger of their right hand on the “J” key. The experiment required participants to make judgments using these keys. First, a “+” sign will appear as a fixation point in the center of the computer screen to signal the start of the experiment. Participants should focus their gaze on a row of arrows displayed on the screen and quickly and accurately determine the direction of the arrows. If the arrow points to the left, press the ‘F’ key; if it points to the right, press the “J” key.

The entire experimental task consists of 304 trials, divided into a practice phase and a test phase. The practice phase comprises 16 trials, during which a cue appears on a gray background for 100 ms, followed by a “+” fixation point for 400 ms. The target stimulus is presented for 1,700 ms, followed by the “+” for 2,000 ms. Participants were instructed to press the button as quickly as possible while ensuring accuracy. After each trial, participants received feedback indicating “correct,” “incorrect,” or “no press” for 500 ms, followed by the reappearance of the “+” fixation point for 400–1,600 ms. The practice phase lasted approximately 2 min. If a participant’s accuracy rate is below 85%, 16 additional practice trials will be conducted; the formal test may not begin until the accuracy rate exceeds 85%. The formal test consists of 288 trials, divided into three test blocks, with each block containing 96 trials: a cue (dual cue or no cue) lasting 100 ms, followed by the presentation of the fixation mark “+” for 400 ms. The target stimulus (target: neutral, congruent, incongruent) appears randomly above or below the “+”; each type of target stimulus is repeated 4 times. The response window is 1,700 ms, followed by the “+” for 2,000 ms, and the “+” fixation mark is presented again for 400–1,600 ms. During the formal test, participants do not receive feedback after making a response. The formal test lasts approximately 10 min, with a mandatory 2-min rest period between blocks (during which participants are instructed to remain quiet, rest with their eyes closed in the same position, and try to clear their minds as much as possible). The alerting effect was measured using reaction time differences; alerting network efficiency is calculated as the reaction time for the no-cue condition minus the reaction time for the dual-cue condition. Since the dual-cue condition provides an additional vigilance cue compared to the no-cue condition, a larger difference between the two indicates higher alerting network efficiency (Yang and Ling, 2019; Fan et al., 2005).

#### 2.2.2 EEG data acquisition and preprocessing

This study utilized the actiCHamp 64-channel EEG acquisition system (Brain Products, Germany). Electrode placement was standardized according to the 10–20 International System, with the FCz point serving as the online reference electrode. The Fpz electrode functioned as the grounding electrode during recording. The EEG parameter settings were as follows: gain = 500, sampling precision = 0.168 μV/LSB, and sampling frequency = 1,000 Hz. The vertical eye movement (VEOG) electrode was positioned 1 cm above the left orbital rim, while the horizontal eye movement (HEOG) electrode was placed 1 cm lateral to the right eye. The electrode cap made direct contact with the subject’s scalp. Conductive gel (Electro-Cap, United States) was used as the conductive medium for collecting scalp EEG signals, and the scalp-electrode contact impedance was maintained below 5 kΩ.

Using the EEGLAB13_0_0b toolbox based on MATLAB 2013b (Delorme and Makeig, 2004), the EEG data were processed in the following order: electrode localization and restoration of the online reference; exclude inactive electrodes, use bilateral mastoid (TP9, TP10) as offline re-referencing, downsampling to 500 Hz; filtering (bandpass filter 0.1–30 Hz), segmentation (−500 to 1,000 ms), and baseline correction (−500 to 0 ms). Subsequently, interpolation and manual rejection were performed for bad segments and bad channels. Following this, Independent Component Analysis (ICA) was conducted (overall rejection rate <10%) (Delorme et al., 2007) to remove noise components (such as blinks, eye drifts, and electromyography), as well as trials with incorrect responses and the immediately following trial, or trials with amplitudes exceeding ±100 μV (overall rejection rate <15%) (Righart and De Gelder, 2008; Wang et al., 2026).

Finally, only trials in which participants responded correctly were selected, and the ERP components for both the dual-cue and no-cue conditions were averaged within each group to obtain the overall average waveforms or topograms for both groups under the dual-cue and no-cue conditions. Based on the objectives of this study and previous research (Hwang et al., 2019; Kao et al., 2019), as well as brain topography and visual detection, the peak amplitude and peak latency of N1 and P2 for each participant under each cue condition were determined. For the N1 component, the central region (average of electrodes CP5, CP3, CP1, CPz, CP2, CP4, and CP6), the parietal region (average of electrodes P7, P5, P3, Pz, P4, P6, and P8 electrodes), the parietal-occipital region (average of electrodes PO7, PO3, POz, PO4, and PO8), and the occipital lobe (average of electrodes O1, Oz, and O3), with a time window of 70–125 ms (Luna et al., 2023; Zhang and Kappenman, 2024). For the P2 component, the frontal lobe (average of AF3, AFz, and AF4 electrodes), the frontal region (average of F3, F1, Fz, F2, and F4 electrodes), the fronto-central region (average of FC3, FC1, FCz, FC2, and FC4 electrodes), the central region (averaged from electrodes C3, C1, Cz, C2, and C4), with a time window of 100–200 ms (Luna et al., 2023; Zhang and Kappenman, 2024; Rong and Che, 2018). The N1 latency is defined as the time interval from the onset of the stimulus to the most negative point in the time-amplitude curve within the 70–125 ms range, and the N1 amplitude is defined as the amplitude at the time of the latency peak. The P2 latency is defined as the time interval from the onset of the stimulus to the most positive point in the time-amplitude curve within the 100–200 ms range, and the P2 amplitude is defined as the amplitude at which the peak occurs.

#### 2.2.3 Statistical analysis

Statistical analysis for this study was performed using SPSS 18.0 (SPSS, Chicago, IL). The K-S method was used to test the normality of the behavioral and EEG data, and the results showed that the behavioral and EEG data for all participants followed a normal distribution (*p* > 0.05). Mixed-design ANOVAs were employed, with the elite athlete group and novice athlete group as between-group factors, and dual-cue and no-cue as within-group factors. For results that did not meet the assumptions of the Greenhouse–Geisser sphericity test, the Greenhouse–Geisser statistic was used to correct for degrees of freedom; the significance level was set at *p* < 0.05. *Post hoc* comparisons were performed using Bonferroni-corrected *t*-tests, and results were reported as means and standard deviations (SD). Further reports included partial eta-squared (*η*^2^) for main effects and interaction effects, as well as estimated effect sizes. The behavioral experiment was designed as a three-factor mixed-design ANOVA with a 2 (group: elite, novice) × 2 (cue: dual-cue, no-cue) × 3 (target: neutral, congruent, incongruent) factorial structure; scores on the alerting network test were analyzed using an independent-samples *t*-test. In addition, ERP analysis was conducted using the amplitude and latency of the N1 and P2 components, employing a three-factor mixed-design ANOVA with the factors 2 (group: elite, novice) × 2 (cue: dual-cue, no-cue) × 4 brain regions. GraphPad Prism 8 and Adobe Illustrator CS5 were used for figure creation.

## 3 Results

### 3.1 Behavioral results

#### 3.1.1 The RT results

**Figure 2:**
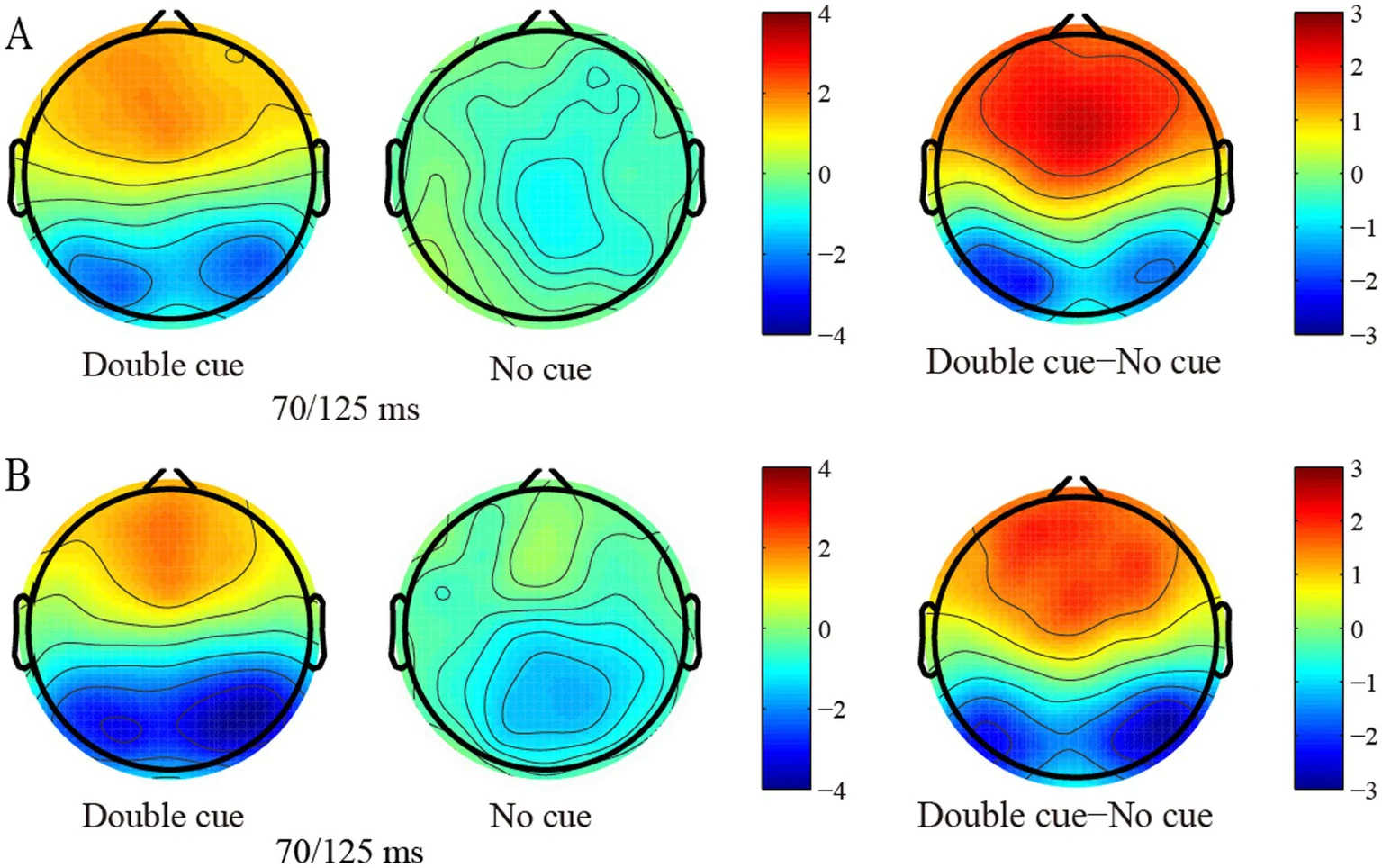
Brain topography of the N1 component for dual cue and no cue. Panel **(A)** represents the EEG topographic map of the N1 (70–125 ms) evoked by the novice group; panel **(B)** represents the EEG topographic map of the N1 (70–125 ms) evoked by the elite group. From left to right are the average brain topographic map for the dual-cue condition, the average brain topographic map for the no-cue condition, and the average difference brain topographic map between the dual-cue and no-cue conditions. The color-coded scale, labeled in μV, indicates activity intensity and is used to interpret the distribution maps.

The RT results show (Table 2 and Figure 2): a significant main effect of group [*F*_(1,62)_ = 19.287, *p* < 0.001, *η*_p_^2^ = 0.237], a significant main effect of cue [*F*_(2,124)_ = 485.166, *p* < 0.001, *η*_p_^2^ = 0.887], a significant main effect of target stimulus [*F*_(1,62)_ = 115.725, *p* < 0.001, *η*_p_^2^ = 0.651], and a significant interaction effect between group and cue [*F*_(2,124)_ = 6.749, *p* = 0.005, *η*_p_^2^ = 0.098]. *Post hoc* analyses revealed that, in between-group comparisons, the elite group had significantly shorter reaction times than the novice group for neutral, congruent, and incongruent target stimuli, all of which were statistically significant (*p* = 0.002, *p* < 0.001, *p* < 0.001, respectively, for no cue; *p* < 0.001 for both cues); within-group comparisons revealed that both the elite and novice groups had significantly longer reaction times for incongruent target stimuli than for neutral and congruent target stimuli, and these differences were statistically significant (*p* < 0.01).

**Table 2:** Responses of various groups to different stimulus types at different cues (*X_* ± SD, ms).

Group	Stimulus type	Cues	
		No cues	Dual cues
Elites	Neutral	492.23 ± 40.72^**^	472.09 ± 31.70^**^
	Congruent	498.87 ± 40.70^**^	481.18 ± 36.39^**^
	Incongruent	568.10 ± 41.00^**^	550.15 ± 39.45^**^
Novices	Neutral	534.02 ± 59.87	520.59 ± 55.66
	Congruent	553.42 ± 61.87	531.13 ± 61.42
	Incongruent	634.38 ± 71.80	618.40 ± 68.51

*Post hoc* comparisons adjusted using the Bonferroni correction: compared with the novice group, ^*^p < 0.05, ^**^p < 0.01. Significance is based on post hoc comparisons following a significant main effect of group in the 2 (group) × 2 (cue) × 3 (target) three-way mixed-design ANOVA.

#### 3.1.2 Alerting network results

The results in Table 3 show that the elite group scored significantly higher than the novice group, and this difference was statistically significant (*t* = −3.659, *p* = 0.001).

**Table 3:** Comparison of scores of two groups of subjects in the alerting network test (*X_* ± SD, ms).

Group	Alerting network
Elites	23.96 ± 15.71^**^
Novices	12.11 ± 9.15

Based on an independent samples *t*-test: compared with the novice group, ^*^p < 0.05, ^**^p < 0.01.

### 3.2 ERP results

#### 3.2.1 N1 peak wave amplitude and latencies

**Figure 3:**
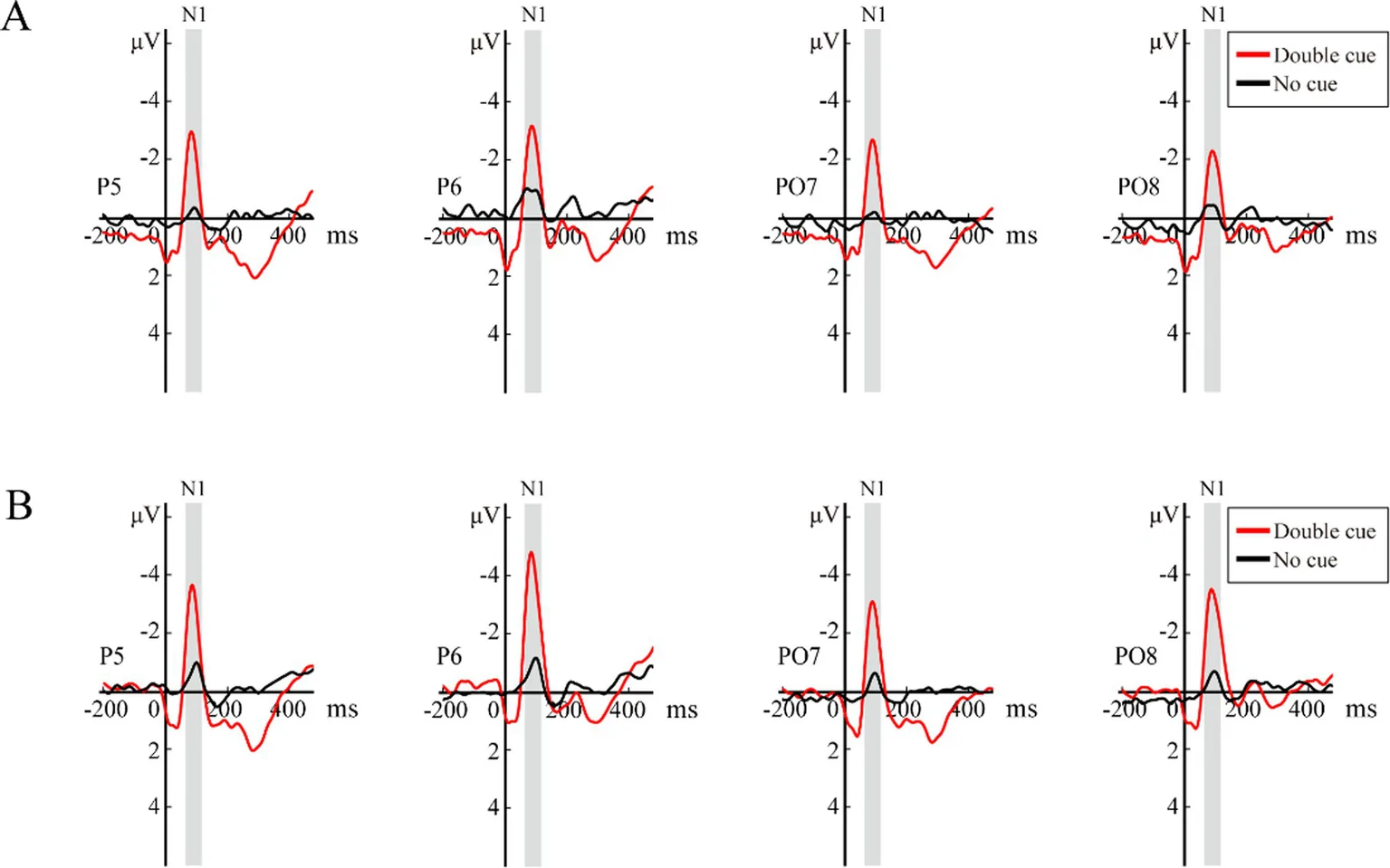
EEG waveforms of the N1 component for dual cue and no cue. Panel **(A)** shows the N1 (70–125 milliseconds) EEG waveforms evoked at P5, P6, PO7, and PO8 in the novice group, while panel **(B)** shows the N1 (70–125 milliseconds) EEG waveforms evoked at P5, P6, PO7, and PO8 in the elite group. The vertical axis of the waveforms represents activity intensity in microvolts (μV), and the horizontal axis represents time in milliseconds (ms). The dark gray shaded area represents the overall average N1 waveform. The red solid line represents the average waveform of the N1 component evoked by dual-cue stimuli, while the black solid line represents the average waveform of the N1 component evoked by no-cue stimuli.

Figures 2, 3 show ERP waveforms under the two conditions: with and without cues. Figure 2 shows a brain topography map, while Figure 3 shows waveform plots; Figure 3A represents the novice group, and Figure 3B represents the elite group.

The results for the N1 peak amplitude (Table 4) showed a significant main effect of group [*F*_(1,62)_ = 8.056, *p* = 0.006, *η*_p_^2^ = 0.115], the main effect of cue was significant [*F*_(1,62)_ = 60.292, *p* < 0.001, *η*_p_^2^ = 0.493], and the main effect of brain region was significant [*F*_(3,186)_ = 16.1566, *p* < 0.001, *η*_p_^2^ = 0.211]. The cue × brain region interaction effect was significant [*F*_(3,186)_ = 32.965, *p* < 0.001, *η*_p_^2^ = 0.347]. *Post hoc* analyses revealed that, in between-group comparisons, the N1 amplitude elicited by the dual-cue condition in the elite group was larger (more negative) than that in the novice group in the central, parietal, parieto-occipital, and occipital regions, and these differences were statistically significant (*p* = 0.020, *p* = 0.003, *p* = 0.011, *p* = 0.41, respectively), whereas for the no-cue condition, a similar pattern was observed only in the central region (*p* = 0.044). Within-group comparisons revealed that for the dual-cue condition in the elite group, the N1 amplitude evoked in the central, parietal, parieto-occipital, and occipital regions was smaller (more negative) than that for the no-cue condition. This difference was statistically significant (*p* = 0.004, *p* < 0.001, *p* < 0.001, *p* < 0.001, respectively), whereas the novice group showed similar findings in the parietal, parieto-occipital, and occipital regions (all *p* < 0.001). We did not find any other main effects or interactions between dual-cue and no-cue conditions regarding N1 amplitude (*F* < 1.796, *p* > 0.05).

**Table 4:** Alerting-induced N1 peak amplitude at different cortical sites (*X_* ± SD, μV).

Group	Cues	Central	Parietal	Parieto-occipital	Occipital
Novices	Dual cue	−1.86 ± 2.20	3.04 ± 1.92^##^	3.23 ± 2.20^##^	−2.19 ± 2.03^##^
Elites	Dual cue	−3.15 ± 2.13^*##^	−4.64 ± 2.25^**##^	−4.76 ± 2.45^*##^	−3.28 ± 2.12^##^
Novices	No cue	1.11 ± 1.63	−1.01 ± 1.71	−0.85 ± 2.13	−0.58 ± 2.09
Elites	No cue	−1.91 ± 1.48	−1.73 ± 1.61	−1.48 ± 1.81	−1.12 ± 1.54

*Post hoc* comparisons based on Bonferroni correction: compared with the novice group, ^*^p < 0.05, ^**^p < 0.01; compared with the no-cue condition, ^#^*p* < 0.05, ^##^*p* < 0.01. Significance is based on *post hoc* comparisons following a significant main effect of group or cue in the 2 (group) × 2 (cue) × 4 (brain region) three-factor mixed-design ANOVA.

On the other hand, regarding the N1 latency induced by cue presentation (Table 5), the results showed a significant interaction effect between cue type and brain region [*F*_(3,186)_ = 13.050, *p* < 0.01, *η*_p_^2^ = 0.174]. We did not find any other main effects or interactions for the N1 latency induced by dual-cue and no-cue conditions (all *F* < 3.135, *p* > 0.05).

**Table 5:** Alerting-induced N1 latency at different cortical sites (*X_* ± SD, ms).

Group	Cues	Central	Parietal	Parieto-occipital	Occipital
Novices	Dual cue	92.64 ± 10.84	94.62 ± 8.11	97.02 ± 9.03	100.59 ± 10.82
Elites	Dual cue	88.27 ± 13.21	91.20 ± 8.16	92.85 ± 9.98	96.29 ± 13.34
Novices	No cue	100.50 ± 14.76	98.91 ± 12.67	95.19 ± 13.44	94.76 ± 15.13
Elites	No cue	98.54 ± 14.61	97.01 ± 12.11	95.30 ± 11.21	96.55 ± 12.24

Based on post hoc comparisons adjusted for the Bonferroni correction, both between-group and within-group comparisons showed *p* > 0.05.

#### 3.2.2 P2 peak waveform amplitudes and latencies

**Figure 4:**
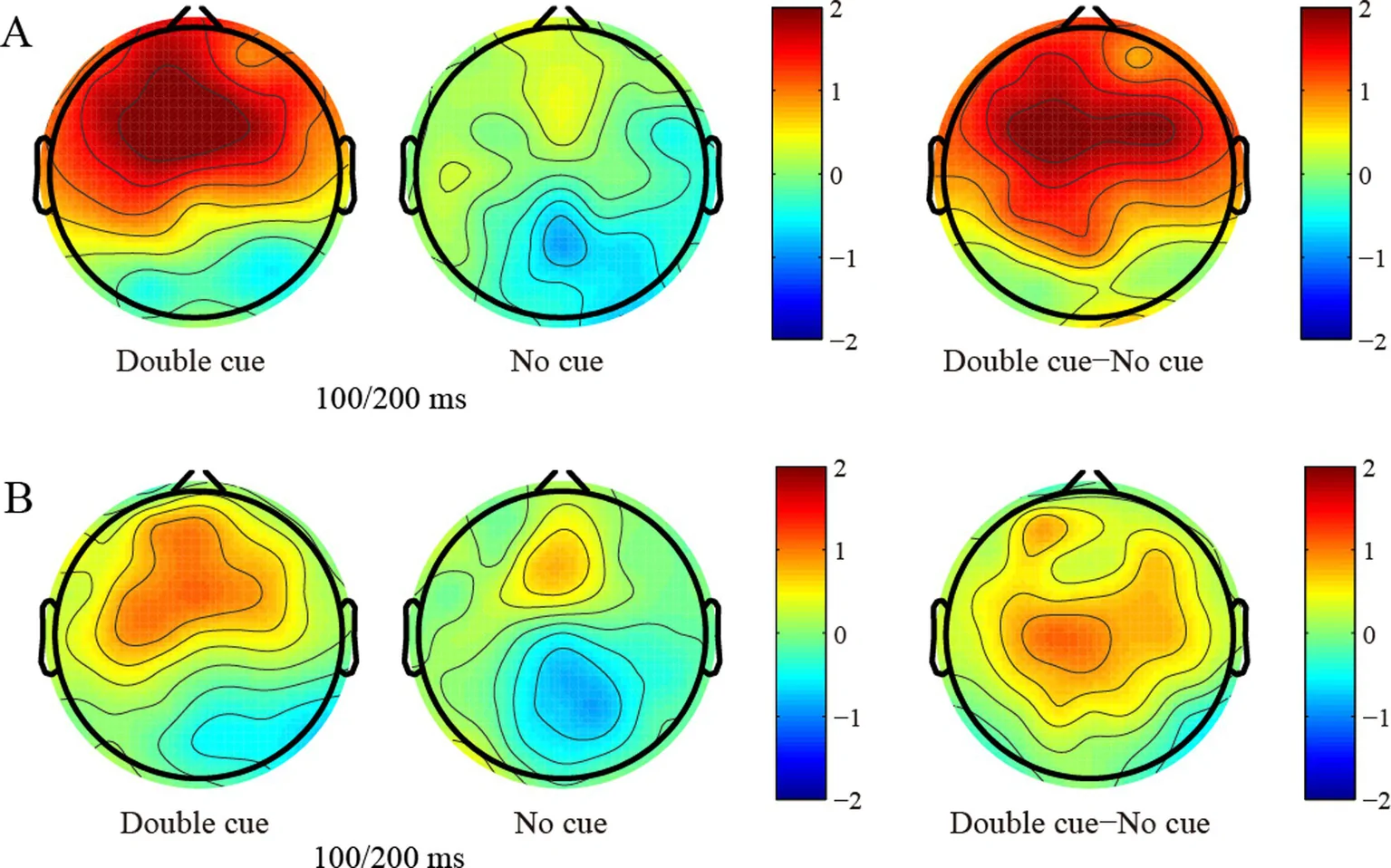
Brain topography of the P2 component for dual-cue and no-cue. Panel **(A)** represents the EEG topographic map of the P2 (100–200 ms) evoked by the novice group; panel **(B)** represents the EEG topographic map of the P2 (100–200 ms) evoked by the elite group. From left to right are the average brain topographic map for the dual-cue condition, the average brain topographic map for the no-cue condition, and the average difference brain topographic map between the dual-cue and no-cue conditions. The color-coded scale, labeled in μV, indicates activity intensity and is used to interpret the distribution maps.

**Figure 5:**
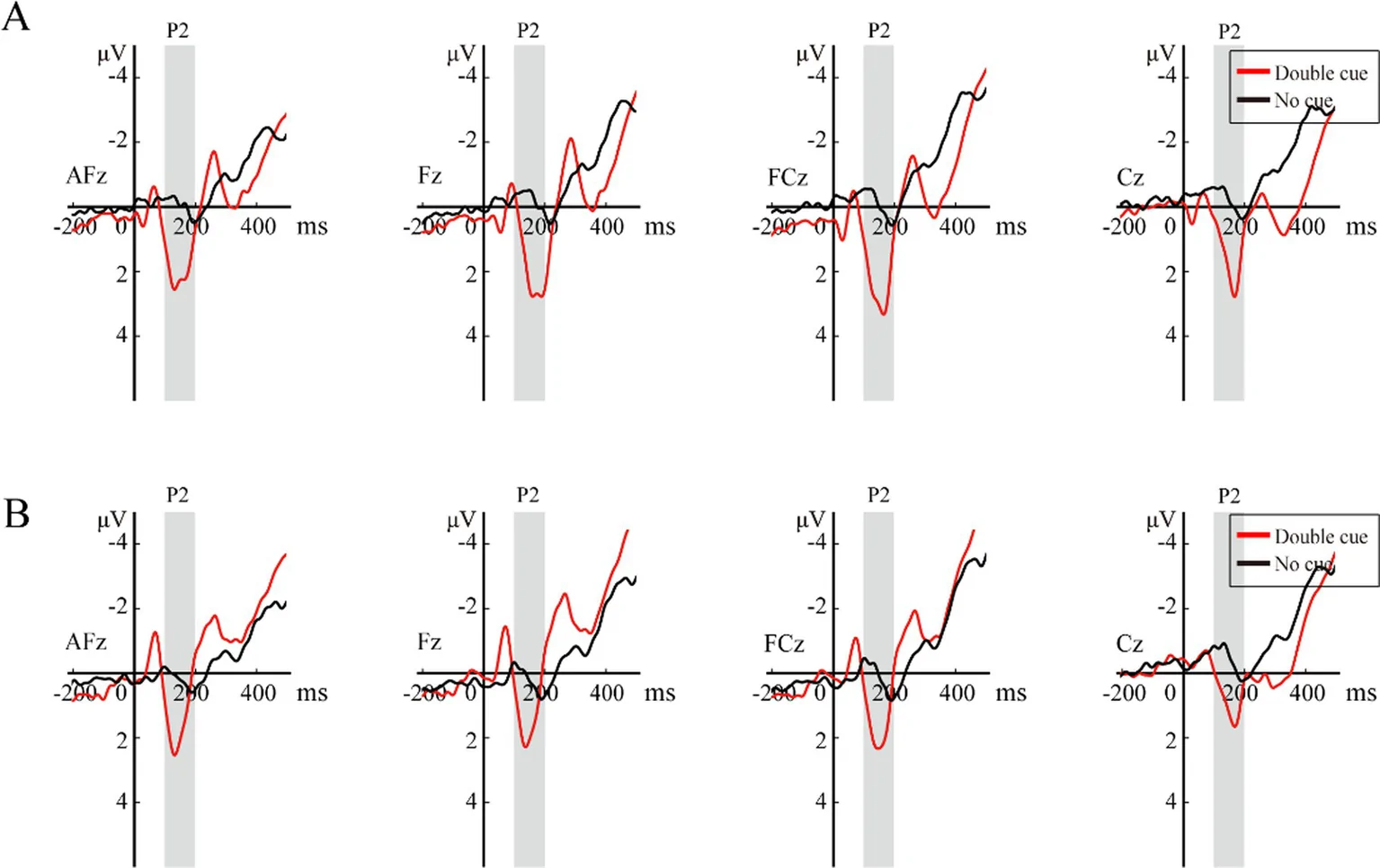
EEG waveforms of the P2 component for dual cue and no cue. Panel **(A)** shows the P2 (100–200 ms) EEG waveforms evoked at AFz, Fz, FCz, and Cz in the novice group, while panel **(B)** shows the P2 (100–200 ms) EEG waveforms evoked at AFz, Fz, FCz, and Cz in the elite group. The vertical axis of the waveforms represents activity intensity in microvolts (μV), and the horizontal axis represents time in milliseconds (ms). The dark gray shaded area represents the overall average P2 waveform. The red solid line represents the average P2 component evoked by dual-cue stimuli, and the black solid line represents the average P2 component evoked by no-cue stimuli.

Figures 4, 5 show ERP waveforms under the two conditions: with and without cues. Figure 4 shows a brain topography map, while Figure 5 shows waveform plots; Figure 5A represents the novice group, and Figure 5B represents the elite group.

The results for P2 amplitude (Table 6) showed a significant main effect of group [*F*_(1,62)_ = 4.325, *p* = 0.042, *η*_p_^2^ = 0.065] and a significant main effect of cue [*F*_(1,62)_ = 79.822, *p* < 0.001, *η*_p_^2^ = 0.563]. *Post hoc* analyses revealed that, in between-group comparisons, the P2 peak amplitude evoked in the frontal, fronto-central, and central regions was larger (more positive) than that in the elite group, and this difference was statistically significant (*p* = 0.022, *p* = 0.016, *p* = 0.014, respectively); however, no similar findings were observed for the no-cue condition (*p* > 0.05). Within-group comparisons showed that in the elite group, the P2 peak amplitude evoked by the dual-cue condition in the prefrontal, frontal, fronto-central, and central regions was larger (more positive) than that evoked by the no-cue condition. This difference was statistically significant (all *p* < 0.001), while a similar pattern was observed in the novice group across the prefrontal, frontal, fronto-central, and central regions (all *p* < 0.001). We found no other main effects or interactions between the dual-cue and no-cue conditions regarding the amplitude of the P2 peak (*F* < 2.246, *p* > 0.05).

**Table 6:** Alerting-induced P2 peak amplitude at different cortical sites (*X_* ± SD, μV).

Group	Cues	Prefrontal	Frontal	Frontal-central	Central
Novices	Dual cue	3.43 ± 2.61^##^	3.98 ± 2.72^##^	4.19 ± 2.75^##^	3.83 ± 2.79^##^
Elites	Dual cue	2.42 ± 1.90	2.61 ± 1.83^*^	2.70 ± 1.94^*^	2.30 ± 1.97^*^
Novices	No cue	0.90 ± 2.59^##^	1.07 ± 2.56^##^	1.11 ± 2.49^##^	1.11 ± 2.18^##^
Elites	No cue	0.47 ± 1.88	0.56 ± 1.63	0.58 ± 1.59	0.40 ± 1.66

*Post hoc* comparisons based on Bonferroni correction: compared with the novice group, ^*^p < 0.05, ^**^p < 0.01; compared with the no-cue condition, ^#^*p* < 0.05, ^##^*p* < 0.01. Significance is based on *post hoc* comparisons following a significant main effect of group or cue in the 2 (group) × 2 (cue) × 4 (brain region) three-factor mixed-design ANOVA.

On the other hand, the P2 latency elicited by cues (Table 7) showed a significant main effect of cue [*F*_(1,63)_ = 56.776, *p* < 0.001, *η*_p_^2^ = 0.474] and a significant main effect of brain region [*F*_(3,189)_ = 16.482, *p* < 0.001, *η*_p_^2^ = 0.207]. *Post hoc* analyses revealed that, within-group comparisons showed that in the elite group, the P2 latency elicited by the dual-cue was significantly earlier than that elicited by the no-cue condition in the prefrontal, frontal, fronto-central, and central regions (*p* < 0.001, *p* < 0.001, *p* < 0.001, *p* = 0.002, respectively), while the novice group also showed similar findings in the prefrontal, frontal, fronto-central, and central regions (*p* < 0.001, *p* < 0.001, *p* < 0.001, *p* < 0.017, respectively). We did not find any other main effects or interactions between dual-cue and no-cue conditions on P2 latency (*F* < 1.597, *p* > 0.05).

**Table 7:** Alerting -induced P2 latency at different cortical sites (*X_* ± SD, ms).

Group	Cues	Prefrontal	Frontal	Frontal-central	Central
Novices	Dual cue	128.59 ± 23.31^##^	130.99 ± 20.53^##^	132.72 ± 21.32^##^	141.91 ± 28.10^##^
Elites	Dual cue	119.21 ± 19.62^##^	122.14 ± 18.69^##^	128.21 ± 21.00^##^	143.01 ± 25.17^##^
Novices	No cue	152.81 ± 30.24	154.74 ± 25.70	157.02 ± 25.46	156.41 ± 27.87
Elites	No cue	154.25 ± 31.07	158.59 ± 26.81	159.35 ± 24.82	162.13 ± 25.01

*Post hoc* comparisons based on Bonferroni correction: compared with the novice group, ^*^p < 0.05, ^**^p < 0.01; compared with the no-cue condition, ^#^*p* < 0.05, ^##^*p* < 0.01. Significance is based on *post hoc* comparisons following a significant main effect of group or cue in the 2 (group) × 2 (cue) × 4 (brain region) three-factor mixed-design ANOVA.

## 4 Discussion

This study revealed the alerting advantages of elite soccer players at both the behavioral and neurophysiological levels. At the behavioral level, the elite group exhibited significantly shorter reaction times than the novice group when performing congruent, incongruent, and neutral stimulus tasks under both dual-cue and no-cue conditions, and their alerting network scores were significantly higher, indicating that elite soccer players have faster reaction times, higher vigilance, and superior alerting network performance; At the ERP level, the elite group exhibited larger N1 peak amplitudes (reflecting enhanced early perceptual gain) and smaller P2 peak amplitudes (reflecting efficient perceptual processing and optimized allocation of cognitive resources) under the vigilance cue condition, providing neurophysiological evidence that long-term training positively shapes the attentional modulation system. These findings are consistent with the existing literature (Mann et al., 2007; Voss et al., 2010; Scharfen and Memmert, 2019). Systematic reviews indicate that athletes generally outperform control groups in cognitive tasks, as evidenced by faster reaction times and higher accuracy rates (Delgado et al., 2025); Large-sample studies have also confirmed that elite athletes’ visuomotor performance is significantly superior to that of less-trained athletes (Burris et al., 2020), and that long-term athletic training is closely associated with improved attentional control efficiency (Pesce et al., 2011). Given the critical role of alert attention in competitive sports, numerous studies have confirmed the positive effects of both chronic and acute exercise on attentional function (Hüttermann et al., 2019; Pesce et al., 2011). Elite soccer players respond more quickly to alerting stimuli than amateur players; this advantage is linked to the positive effects of soccer training on the alerting network (Verburgh et al., 2014), and sports with high perceptual and decision-making demands are positively correlated with an individual’s baseline level of alertness (Ballester et al., 2019). The efficient cognitive processing capabilities demonstrated by elite athletes help enhance decision-making speed and judgment accuracy in sports contexts.

This study shows that vigilance cues enhanced the amplitude of the N1 peak in the posterior brain, particularly in the parietal and parieto-occipital regions. The modulatory effect of cue-induced selective attention on N1 amplitude is consistent with previous findings regarding the alerting effect in the parietal cortex (Fan et al., 2007). Luque-Casado et al. (2013) found that during a 10-min vigilance task, the high-fitness group (VO₂max = 69.05 ± 5.6 mL·kg^−1^·min^−1^) exhibited superior vigilance compared to the low-fitness group (VO₂max = 36.19 ± 5.5 mL·kg^−1^·min^−1^). The cue-N1 amplitude under dual-cue conditions was significantly greater than that under no-cue conditions, reflecting a phasic alerting effect; simultaneously, the modulatory effect of the cue on the target stimulus indicated that participants maintained a sustained alerting effect toward subsequent target stimuli (Chacko et al., 2020). This study found that elite soccer players exhibited relatively high reaction readiness efficiency, consistent with behavioral data, reflecting high levels of vigilance and the ability to effectively shift attention guided by spatial cues. The N1 component is considered to reflect early attentional processing of sensory information—its amplitude is typically larger when the target stimulus is noticed (Williams et al., 2016). Under both cue conditions, elite athletes exhibited larger N1 peak amplitudes, which is consistent with previous research findings (Fu et al., 2008). Increased N1 peak amplitude indicates an enhanced flow of perceptual information, reflecting directed and intensified processing of input information during primary sensory analysis (Näätänen and Michie, 1979)—that is, the mobilization of more attentional resources to focus on the impending target stimulus. It is worth noting that although this study observed a significant increase in N1 amplitude among elite soccer players, no significant between-group differences were found in N1 latency. This dissociation between amplitude and latency has important theoretical implications. The N1 latency is thought to reflect the temporal processes of early sensory coding and feature extraction, and is more sensitive to the physical properties of the stimulus (such as intensity and modality) and the conduction efficiency of sensory pathways (Näätänen and Picton, 1987; Luck, 2014), whereas N1 amplitude more strongly reflects the intensity of top-down modulation of attentional resources directed toward the sensory cortex (Hillyard and Anllo-Vento, 1998). Therefore, the pattern observed in this study—where amplitude was enhanced while latency remained unchanged—suggests that the vigilance advantage of elite soccer players is primarily manifested as the optimized allocation of attentional resources guided by spatial cues and enhanced perceptual gain, rather than a general acceleration of conduction speed in primary sensory pathways. In other words, the cognitive advantages shaped by long-term athletic training are more likely to act on attentional modulation systems (such as the resource allocation mechanisms of the parietal–frontal alerting network) rather than altering the fundamental temporal characteristics of lower-level visual pathways. This explanation is consistent with the pattern of results showing enhanced N1 amplitude but unchanged latency in athletes, and it aligns with the theoretical framework of “perceptual-attentional dissociation”—namely, that attention can modulate the intensity (amplitude) of sensory processing without necessarily altering its temporal course (latency) (Mangun and Hillyard, 1991; Luck and Hillyard, 1994). Furthermore, the reduction in reaction time observed at the behavioral level, given the absence of changes in N1 latency, should be attributed to enhanced reaction readiness or accelerated late-stage reaction selection triggered by heightened arousal, rather than a compression of early sensory encoding time. This distinction also suggests that future research could incorporate earlier components (such as P1) or employ time-frequency analysis (such as alpha-band desynchronization) to further clarify the specific effects of motor expertise on attentional modulation across different temporal windows. Regarding the practical implications of these ERP differences for soccer performance, existing evidence indicates that N1 enhancement may be directly linked to several key abilities in actual matches. First, the early selective attentional advantage reflected by increased N1 amplitude in elite soccer players has been shown in action anticipation tasks to facilitate the rapid integration of *a priori* information with kinematic information, thereby improving the accuracy of anticipatory judgments (Ji et al., 2023). Second, studies using drift-diffusion models have revealed that the vigilance advantage of elite soccer players stems from reduced latencies in information encoding and motor generation, and that this advantage is closely related to the structural-functional connectivity of specific subregions of the thalamus (Zhong et al., 2025), suggesting that the enhanced early perceptual processing reflected by the N1 has a genuine neurostructural basis. Furthermore, studies on complex sports training interventions have confirmed that improvements in sustained attention and inhibitory control can be captured through ERP measures, and that these improvements directly translate into enhanced reaction speed and accuracy at the behavioral level. Other research has indicated that sport-specific cognitive experience (rather than mere physical fitness) is a key predictor of vigilance performance and inhibitory control (Ballester et al., 2019). Taken together, the enhanced N1 amplitude observed in elite soccer players in this study may reflect perceptual gains and the ability to optimize the allocation of attentional resources shaped by long-term training. Under the high-time–pressure conditions of soccer matches, these abilities manifest as faster reaction times, more precise anticipatory judgments, and more stable maintenance of alertness, thereby constituting an important cognitive-neural foundation for the competitive performance advantage of elite athletes.

This study also found that, under both dual-cue and no-cue conditions, the elite group exhibited smaller P2 peak amplitudes, and this effect was primarily observed in relevant leads such as the frontal, fronto-central, and central regions. Additionally, it was found that, compared to the no-cue condition, the dual-cue condition elicited larger P2 peak amplitudes and shorter latencies. It should be noted that the functional significance of the P2 component remains somewhat heterogeneous in the existing literature, and the interpretation of whether an increase or decrease in its amplitude indicates a positive or negative effect depends heavily on the specific experimental paradigm and cognitive demands (Luck, 2014; Crowley and Colrain, 2004). In tasks involving emotional stimuli, conflict monitoring, or high cognitive load, an increase in P2 amplitude is typically interpreted as enhanced encoding of task-relevant features or heightened alertness (Carretié et al., 2001; Potts, 2004); whereas in paradigms emphasizing perceptual efficiency or automated processing, a reduction in P2 amplitude may reflect efficient perceptual processing or the economical allocation of neural resources (Shi et al., 2024; Xiong and Song, 2024). For example, Qi et al. (2017) found in a Go/Nogo task under stress conditions that a reduction in P2 amplitude was accompanied by an increase in N2 amplitude, which they interpreted as a dynamic reallocation of early selective attentional resources toward later cognitive control processes; Bertsch et al. (2011) also reported that P2 amplitude tended to decrease when reaction times were faster and accuracy declined, suggesting that P2 is sensitive to dynamic changes in attentional resource allocation. The above studies indicate that the direction of change in P2 amplitude does not inherently carry a single “good/bad” value attribute but should be interpreted within the context of specific task conditions. Within the framework of the alerting task in this study—which requires participants to continuously monitor targets and respond rapidly to cue signals—the reduced P2 peak amplitude observed in elite soccer players is more likely to be interpreted as a feature of efficient perceptual processing shaped by long-term soccer training. The combination of task stimuli and motor demands in this study helped modulate activity in the prefrontal cortex and anterior cingulate cortex, suggesting enhanced selective attention (Wu et al., 2019). Changes in P2 amplitude reflect the influence of cue location on perceptual processing, indicating a facilitative effect at the level of cue location processing and a correlation with enhanced ability to control attentional allocation (Zhou et al., 2020). Regarding the potential impact of this ERP difference—a reduced P2 peak amplitude in elite soccer players—on soccer performance, existing evidence suggests that it may reflect mechanisms of optimized perceptual processing shaped by long-term training and is directly related to multiple key abilities in actual matches. First, Xiong and Song’s (2024) study on high-pressure decision-making in elite athletes confirmed that they exhibit reduced P2 peak amplitude during the action selection phase. This reduction occurs in conjunction with increased N1 amplitude and reduced P3 amplitude, reflecting the optimized allocation of cognitive resources during early stages of perceptual processing—that is, the efficient suppression of task-irrelevant distractor information under task demands, thereby focusing attention on key stimulus features. A mechanism that is particularly critical in high-time–pressure situations during soccer matches. Second, a study by Wang et al. (2022) on tennis players found that elite athletes had significantly shorter P2 latencies in the occipital region compared to novices, indicating that they direct attention to the features of moving objects earlier during the early perceptual processing stage, suggesting that P2 may serve as a potential neural biomarker for assessing sport-specific perceptual abilities. Furthermore, a study by Shi et al. (2024) on table tennis players showed that elite athletes had significantly lower P2 amplitude in the occipital region compared to the control group, which was attributed to the neural efficiency hypothesis—long-term specialized training enables athletes to perform efficient perceptual processing with less neural resource consumption. In summary, the smaller P2 peak amplitude observed in elite soccer players in this study under both dual-cue and no-cue conditions may reflect the enhanced efficiency of perceptual processing accumulated through long-term training. Specifically, specialized training has shaped a more efficient early perceptual processing pattern, manifested as faster visual search speed, earlier attentional engagement, and more effective suppression of irrelevant information. In the dynamic environment of a soccer match, this advantage in early perceptual processing can be directly translated into faster reaction times, more accurate situational judgment, and more stable sustained alerting, thereby constituting an important cognitive-neural foundation for the competitive performance advantage of elite soccer players.

In summary, soccer is an open-skill sport that involves multiple teammates, opponents, and tactical strategies. During training or competition, athletes must maintain a state of constant alertness through their attentional networks in order to be prepared to receive and process various complex pieces of information. This study found that elite soccer players exhibited stronger alertness responses to arousal cues, and behavioral data also confirmed significantly shorter reaction times during rest. Previous research has shown that elite athletes differ significantly from novices or amateur non-athletes in terms of attentional network performance (Montuori et al., 2019), and that soccer players have superior visual attention abilities compared to non-athletes (Faubert, 2013). These results suggest that elite soccer players may possess a stronger ability to induce endogenous arousal in the absence of external cues. The advantage of the alerting network reflected in soccer training can be assessed through the peak amplitude and latency of ERP components (N1 and P2), thereby providing neurophysiological evidence for the future development of an evaluation system to enhance training performance in soccer.

## 5 Limitations

Admittedly, this study has several limitations. First, the cross-sectional design limits our inferences regarding temporal dynamics and causal relationships. Future research could adopt longitudinal tracking or experimental manipulation paradigms to further elucidate the dynamic evolution of the alertness effect in sports contexts. Second, this study focused solely on the alertness performance of elite soccer players and did not include physical fitness indicators (such as maximum oxygen uptake, training load, and daily physical activity levels). Future studies should comprehensively consider these factors to clarify whether the observed effects stem from sport-specific experience or from broader physical fitness advantages. Furthermore, the sample was limited to elite soccer players, which to some extent restricts the generalizability of the conclusions to other athlete populations. Future research should further test the replicability of these findings across different sports, genders, and playing positions, and may include non-athlete control groups to enhance external validity. Finally, although the alerting task used in this study is well-suited for this purpose, sports training may exert differential effects on different subsystems of the attentional network (such as the orienting network and the executive control network), and these effects may vary depending on the assessment tool used. Therefore, future studies could employ multiple task paradigms to systematically compare different types of alertness effects, thereby providing a more comprehensive picture of the multidimensional relationship between sports interventions and attentional function. Furthermore, this study primarily relied on ERP time-domain measures for analysis, whereas the neural mechanisms underlying the alertness effect inherently involve multi-frequency oscillations and coordinated activity across multiple brain regions. Future research could further incorporate complementary neurophysiological methods, such as: employing time-frequency analysis (e.g., changes in power in the theta and alpha bands) to characterize oscillatory dynamics during alerting; or employing functional connectivity analysis (such as phase synchronization or coherence) to investigate the long-range interaction patterns of the frontoparietal network during the alerting process; multimodal imaging techniques, such as fMRI and fNIRS, could also be integrated to elucidate the neural basis of the alertness effect and its plasticity mechanisms in elite athletes with higher spatiotemporal resolution.

## 6 Conclusion

This study systematically examined the alertness advantages of elite soccer players from both behavioral and neurophysiological perspectives. Behavioral results showed that, under both dual-cue and no-cue conditions, the elite group had significantly shorter reaction times than the novice group when performing congruent, incongruent, and neutral stimulus tasks, and their alerting network scores were significantly higher than those of the novice group, indicating that elite soccer players possess faster reaction speeds and higher levels of vigilance. ERP results showed that the elite group exhibited larger N1 peak amplitudes and smaller P2 peak amplitudes under the alertness cue condition. Specifically, the enhanced N1 amplitude reflects increased early perceptual gain and the effective allocation of attentional resources to the sensory cortex, while the reduced P2 amplitude reflects the efficiency in perceptual processing and the optimized allocation of cognitive resources shaped by long-term training. Notably, no significant between-group differences were found in N1 latency. This dual-separation pattern of amplitude and latency suggests that the alertness advantage of soccer players is primarily manifested as improved efficiency in the allocation of resources within attentional modulation systems (such as the parietal–frontal alerting network), rather than a general acceleration of conduction speed in primary sensory pathways.

The scientific significance of this study is reflected in the following aspects. At the theoretical level, the study provides new evidence from the alerting network supporting the notion that “long-term sports training can shape specific cognitive functions,” thereby enriching cognitive neuroscience theories in the field of sports expertise. It also reveals the complementary nature of N1 and P2 amplitudes in reflecting different dimensions of alertness function—the former focusing on the intensity of perceptual gain, and the latter on the efficiency of perceptual processing. At the methodological level, the study validated the sensitivity and effectiveness of ERP time-domain analysis (N1/P2 components) in assessing athletes’ alertness function, providing quantifiable electrophysiological indicators for future research. At the practical level, the results suggest that changes in N1 and P2 amplitudes may serve as potential neural biomarkers for evaluating athletes’ alertness states and training adaptability, offering scientific guidance for athlete selection and the development of personalized training programs.

In summary, this study confirms that elite soccer players possess dual advantages in alertness function at both the behavioral and neurophysiological levels. It provides new empirical evidence for understanding the neuroplasticity mechanisms by which long-term athletic training shapes cognitive function, and offers valuable insights for the fields of sport cognitive neuroscience and sports science practice.

## Data Availability

The original contributions presented in the study are included in the article/supplementary material, further inquiries can be directed to the corresponding author.
